# Successful Response to Intravitreal Faricimab Injections in a Case of Neovascular Age-Related Macular Degeneration in Vitrectomized Eyes

**DOI:** 10.7759/cureus.82709

**Published:** 2025-04-21

**Authors:** Shinichiro Chujo, Hisashi Matsubara, Yoko Mase, Kumiko Kato, Mineo Kondo

**Affiliations:** 1 Department of Ophthalmology, Mie University Graduate School of Medicine, Tsu, JPN

**Keywords:** avitreous, faricimab, intravitreal injection, neovasucular age related macular degeneration, oct (optical coherence tomography), vitrectomy, retina

## Abstract

The half-life of anti-vascular endothelial growth factor (anti-VEGF) drugs shortens significantly after vitrectomy due to an increased intraocular pharmacokinetic clearance caused by the absence of the vitreous gel. This reduced half-life can limit the therapeutic effect of these intravitreal agents and complicate the management of conditions such as neovascular age-related macular degeneration (nAMD). We report a case of nAMD in a vitrectomized eye that showed inadequate response to both aflibercept and brolucizumab, but experienced effective suppression of exudation following intravitreal administration of faricimab. This case suggests that faricimab may offer a therapeutic benefit in managing exudative changes in vitrectomized eyes with nAMD.

## Introduction

The incidence of neovascular age-related macular degeneration (nAMD) is increasing worldwide [1]. The first-line treatment for nAMD typically involves intravitreal injections of anti-vascular endothelial growth factor (anti-VEGF) agents [2], administered following a Treat-and-Extend (TAE) regimen [3]. Although the TAE regimen has been reported to maintain long-term visual acuity, the treatment often needs to be continued over an extended period [4]. As long-term repeated injections can burden patients, treatment discontinuation has been proposed in some cases [5].

However, our group reported that approximately 50% of the cases had a recurrence within one year, and approximately 73.9% had a recurrence within five years after discontinuation of the anti-VEGF intravitreal injections. This led to the understanding that long-term anti-VEGF therapy is essential to prevent the recurrence of neovascularization [6].

Following vitrectomy, the replacement of the vitreous gel with aqueous humor leads to an accelerated intraocular drug clearance, thereby shortening the intravitreal half-life of anti-VEGF agents [7,8]. This can decrease their long-term effectiveness and make the treatment of nAMD and other conditions transient.

We report a case of nAMD in an eye that had undergone vitrectomy with the replacement of the vitreous gel. It did not respond to treatment with either aflibercept or brolucizumab. However, there was a good suppression of exudation after intravitreal injections of faricimab. Significantly, the exudation remained suppressed even for a long time after treatment discontinuation.

## Case presentation

This case is of an 82-year-old male patient. He was referred to our hospital from a private clinic for the treatment of a displaced intraocular lens (IOL) in his right eye.

**Figure 1:**
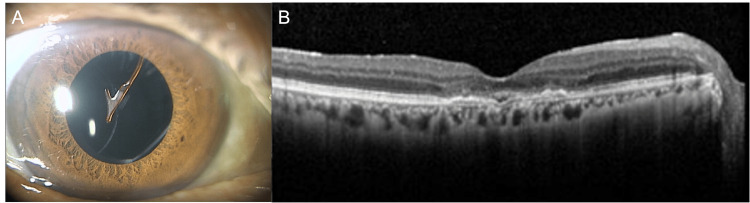
Slit-lamp and optical coherence tomographic (OCT) images of the patient's right eye with neovascular age-related macular degeneration (nAMD) A: IOL dislocation is observed. B: At the time of the initial examination, the macula was dry. IOL, intraocular lens

He had previously been diagnosed with nAMD in the right eye and had been receiving fixed doses of aflibercept for eight weeks, with good fluid control. The best-corrected visual acuity (BCVA) was 20/25 and intraocular pressure was 13 mmHg in the right eye. Slit lamp examination revealed IOL deviation in the same eye. Optical coherence tomography (OCT) showed that it had no exudative changes and a dry macula was maintained (Figures 1A, 1B)

**Figure 2:**
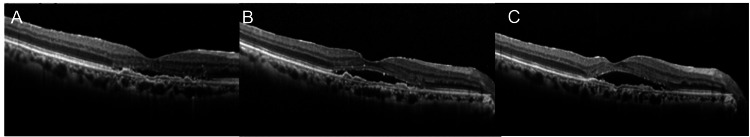
OCT image during the follow-up period after vitrectomy A: OCT findings four weeks after the surgery; SRF observed. B: OCT findings four weeks after the first administration of aflibercept; SRF persists. C: OCT findings four weeks after the second administration of aflibercept; SRF persists. OCT, Optical coherence tomography; SRF, subretinal fluid

The patient underwent vitreous surgery and IOL scleral fixation. The postoperative course was good, but the exudative changes recurred about four weeks after the surgery (Figure 2A). Therefore, we started intravitreal injections with aflibercept (IVA) again, but the subretinal fluid (SRF) recurred within eight weeks (Figures 2B, 2C)

**Figure 3:**
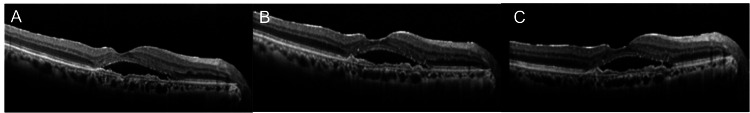
OCT results after switching to ranibizumab A: OCT image before ranibizumab administration; SRF present. B: OCT image four weeks after ranibizumab administration; SRF persists. C: OCT image four weeks after switching back to aflibercept; SRF persists. OCT, optical coherence tomography; SRF, subretinal fluid

The patient reported no subjective change in symptoms during this period. We then switched to ranibizumab, considering the possibility of tachyphylaxis to aflibercept, but there was no improvement in SRF at four weeks after the injection (Figures 3A-3C).

**Figure 4:**
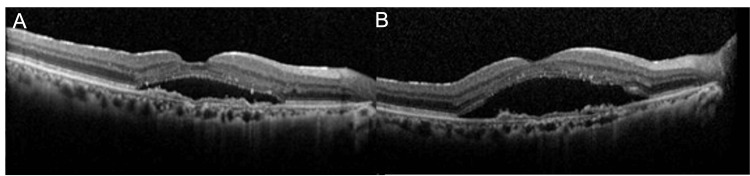
OCT results after switching to brolucizumab A: OCT image before brolucizumab administration; SRF observed. B: OCT four weeks after brolucizumab administration; SRF persists. OCT, optical coherence tomography; SRF, subretinal fluid

The possibility of tachyphylaxis to aflibercept as a cause of the poor response was ruled out. We then switched to brolucizumab, but there was no improvement in SRF at four weeks after the injection (Figures 4A, 4B).

**Figure 5:**
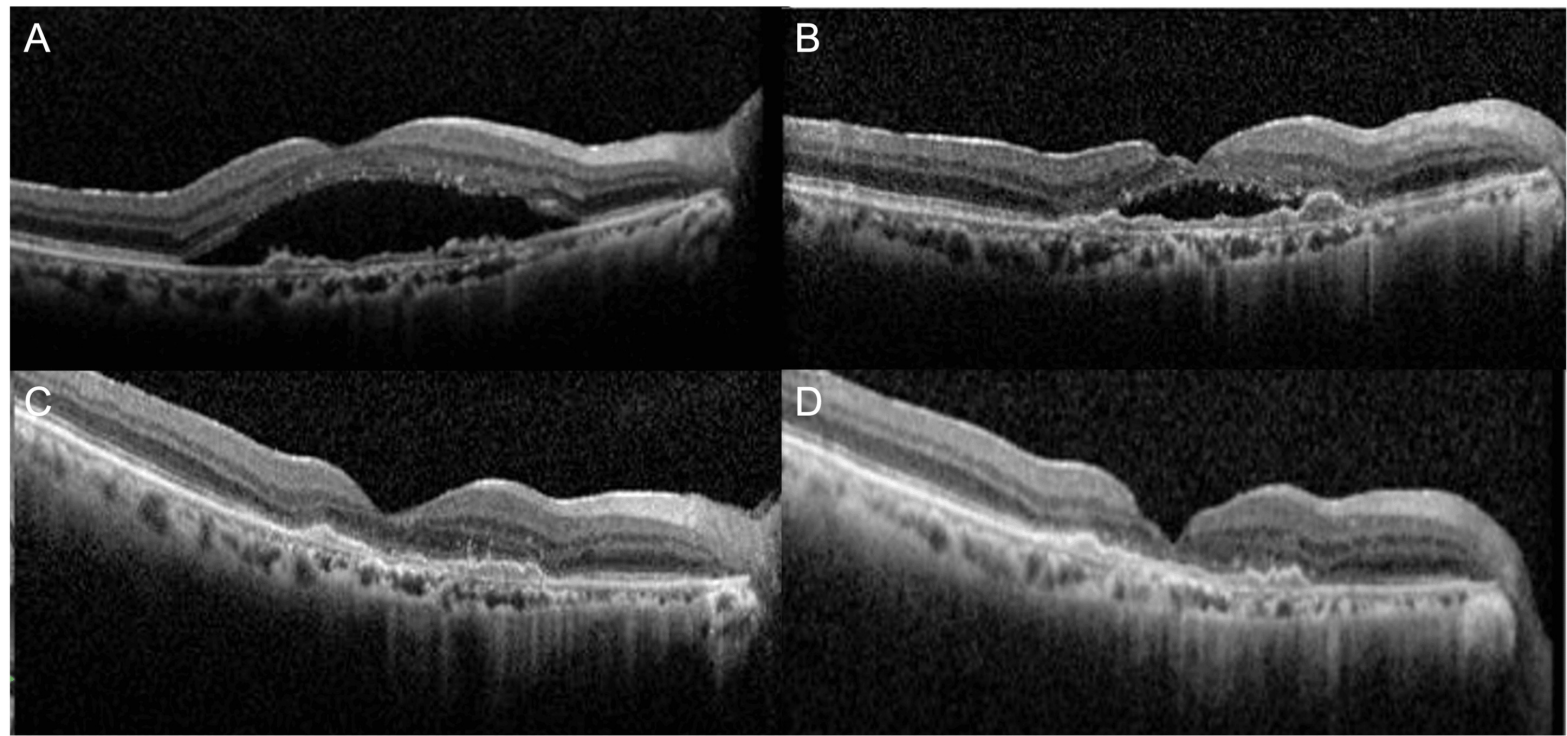
OCT findings after switching to faricimab A: OCT before administration B: OCT four weeks after the first administration of faricimab; a decrease in SRF observed. C: OCT four weeks after the second administration of faricimab; disappearance of SRF observed. D. OCT four weeks after the third administration of faricimab; SRF remains absent. OCT, optical coherence tomography; SRF, subretinal fluid

As brolucizumab was also ineffective, photodynamic therapy was considered as a treatment option. However, after discussion with the patient, it was decided to proceed with faricimab administration. Hence, we switched to faricimab, with another loading therapy (Figure 5A). After the first intravitreal injection of faricimab, a slight improvement in SRF was observed (Figure 5B). By the second intravitreal injection, SRF had disappeared (Figure 5C). And after the loading therapy was over, the patient's macula became dry (Figure 5D).

**Figure 6:**
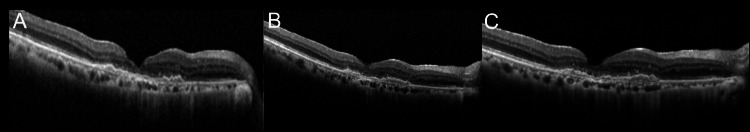
OCT after discontinuation of treatment A: OCT image before discontinuation of treatment. The dry macula is maintained. B: OCT image at the first visit after discontinuation of treatment. The dry macula is maintained. C: OCT image one year after discontinuation. OCT, optical coherence tomography

The patient was scheduled to receive maintenance therapy, but then liver cancer was detected, and he stopped attending the hospital for about eight months while undergoing chemotherapy. However, the dry macula was maintained (Figures 6A, 6B).

At this stage, we had a thorough discussion with the patient about whether to follow a fixed dosing regimen or to adopt a pro re nata (PRN) approach based on the disease progression. The patient wished to continue with the latter, so we continued to monitor his condition. As of now, a year has passed since the last injection. The dry macula was maintained (Figure 6C) and the BCVA was 20/60 in the right eye.

A summary of the treatment course and the therapeutic responses is presented in Table 1 to facilitate understanding of the patient's clinical course.

**Table 1:** Summary of the therapeutic agents used and the treatment course SRF, subretinal fluid

Timeline	Drug	Interval	OCT Findings	Response
Pre-vitrectomy	Aflibercept	Fixed dose 8 weeks	Dry macula	Good response
4 weeks post-op	–	–	SRF recurrence	–
Restart Injection	Aflibercept	Once	SRF persists at 4 & 8 weeks	No response
Switch #1	Ranibizumab	Once	SRF remains at 4 weeks	No response
Switch #2	Aflibercept	Once	SRF remains at 4 weeks	No response
Switch #3	Brolucizumab	Once	SRF remains at 4 weeks	No response
Switch #456 (Loading)	Faricimab	Once a month for 3 months	SRF reduction, dry macula after the second dose	Good response
8 months follow-up	–	–	Dry macula maintained	Maintained
12 months follow-up	–	–	Dry macula maintained	Maintained

## Discussion

We have presented our findings in one case of nAMD in which the exudative activity was not controlled by brolucizumab and only by intravitreal injections of faricimab. Interestingly, the disease activity was controlled for at least a year. In general, reports suggest that when anti-VEGF drugs are suspended, the disease recurs within a year in more than 50% of the cases [9]. In our case, no recurrence was observed for approximately one year after the intravitreal injection of faricimab was stopped.

Intravitreal injections of brolucizumab failed to achieve adequate fluid control, whereas initiating loading therapy with faricimab resulted in good fluid resolution. This improvement is likely due to faricimab’s enhanced ability to suppress nAMD activity. The underlying reason may be linked to the altered clearance of anti-VEGF agents in eyes lacking the vitreous gel. A report by Lee et al. has shown that the effect of anti-VEGF drugs is reduced in an eye with a vitreous replacement after vitrectomy, likely due to accelerated drug clearance in the saline-filled cavity [10].

It has also been reported that the pharmacokinetic half-life of anti-VEGF drugs varies indirectly with the molecular weight of the drug [10]. In non-vitrectomized eyes, the molecular weights of aflibercept, brolucizumab, and ranibizumab are approximately 115 kDa, 26 kDa, and 48 kDa, respectively [11-13]. Faricimab (149 kDa) may benefit from its relatively large molecular size, potentially reducing clearance in vitrectomized eyes [14]. Thus, we can consider the possibility that faricimab, with its large molecular weight, was effective in an eye where the vitreous gel was replaced by saline, leading to decreased clearance.

We also considered the possibility that the long-term suppression of disease activity observed with faricimab loading therapy may have been influenced by prior disease control achieved through the fixed eight-week aflibercept administration before surgery. Kataoka et al. reported that switching to faricimab was effective in patients with nAMD who were unable to extend the dosing interval with aflibercept, showing positive outcomes six months after the switch [15]. Similarly, Machida et al. demonstrated that faricimab was particularly effective in cases characterized by a thin choroid, non-polypoidal choroidal vasculopathy (non-PCV), and a short dosing interval prior to switching [16]. In addition to its higher molecular weight, faricimab’s dual inhibition of VEGF-A and Angiopoietin-2 may have contributed to sustained disease control by enhancing vascular stability and reducing inflammation, which could be especially beneficial in vitrectomized eyes [14].

As indicated by these reports, there are cases of nAMD in which a switch to faricimab is effective. This patient also showed the presence of a thin choroid. At the initial visit, the subfoveal choroidal thickness was 181 μm, which is thinner than that observed in the general population (283.7±84.1 μm) [17]. We hence consider the possibility that a switch to faricimab was effective.

The findings of this case report are limited by its single-patient design. Additional studies with larger sample sizes are needed to further explore and substantiate the outcomes observed.

Our findings indicate that faricimab may be effective in cases of nAMD in vitrectomized eyes. However, further studies with a larger patient population are needed to validate these results.

## Conclusions

We reported a case of nAMD in a vitrectomized eye where exudative changes were successfully suppressed over the long term following a switch to faricimab. The patient had shown poor response to previous treatments with aflibercept and brolucizumab, but faricimab led to resolution of subretinal fluid and stabilization of the retinal morphology.

This case suggests that faricimab, a dual inhibitor of VEGF-A and Angiopoietin-2 with a relatively large molecular weight, may be particularly effective in eyes that have undergone vitrectomy, where intravitreal drug clearance is accelerated. Although limited by being a single case report, the present findings support the need for further investigation into the pharmacologic advantages of faricimab in vitrectomized eyes with treatment-resistant nAMD.
